# Strong nuclear EGFR expression in colorectal carcinomas is associated with cyclin-D1 but not with gene EGFR amplification

**DOI:** 10.1186/1746-1596-6-108

**Published:** 2011-11-03

**Authors:** Andrea Dekanić, Renata Dobrila Dintinjan, Ivana Budisavljević, Sanja Pećanić, Marta Žuvić Butorac, Nives Jonjić

**Affiliations:** 1 Department of Pathology, School of Medicine, University of Rijeka, 51000 Rijeka, Croatia; 2 Department of Radiotherapy and Oncology, University Hospital Rijeka, 51000 Rijeka, Croatia; 3 Faculty of Engineering, University of Rijeka, 51000 Rijeka, Croatia

**Keywords:** colorectal carcinoma, nuclear EGFR, cyclin-D1

## Abstract

**Background:**

Prognostic and predictive significance of epidermal growth factor receptor (EGFR) in colorectal carcinomas (CRCs) is still controversial. The aim of the present study was to explore and correlate membrane and nuclear EGFR and cyclin-D1 protein expression with EGFR gene status of tumor cells.

**Methods:**

Immunohistochemical and FISH analysis was performed on 135 archival formalin fixed and paraffin embedded CRCs.

**Results:**

Strong membrane and strong nuclear EGFR staining was detected in 16% and 57% of cases, respectively, and strong cyclin-D1 expression in 57% samples. Gene EGFR amplification was identified in 5.9% and polysomy in 7.4% of cases, while 87% showed no EGFR gene changes. A statistically significant difference was only found between tumor grade and expression of membrane EGFR, while nuclear EGFR and cyclin-D1 expression was not associated with the clinicopathologic characteristics analyzed. Tumor cells displaying gene amplification and strong protein membrane EGFR expression overlapped, while EGFR gene status showed no correlation with nuclear EGFR and cyclin-D1. There was no association between membrane EGFR and cyclin-D1, whereas nuclear EGFR expression was strongly related to cyclin-D1 expression.

**Conclusions:**

Study results revealed heterogeneity among CRCs, which could have a predictive value by identifying biologically and probably clinically different subsets of tumors with the possibly diverse response to anti-EGFR therapies.

## Background

Colorectal cancer (CRC) is the second most common cause of cancer-related death in developed countries, as in the last few years the incidence has been increasing with decreasing age at diagnosis. The 5-year relative survival rate is approximately 45%, demonstrating an improvement from 30 years ago, when the survival rate was 30% [1]. The irinotecan and oxaliplatin have improved survival while the development of monoclonal antibodies against growth factor receptors, such as monoclonal antibodies against epidermal growth factor receptor (EGFR), has augmented their effects [2,3].

EGFR (ErbB1) is a glycoprotein composed of an extracellular ligand-binding domain, a transmembrane region, and an intracellular tyrosine kinase domain. The receptor is a member of the ErbB family of receptor tyrosine kinases, including ErbB1, ErbB2 (HER-2), ErbB3, and ErbB4, and it is encoded by the c-*erb*B protooncogene. In normal and malignant cells, the activation of EGFR receptor cascades has multiple consequences, such as cell growth, differentiation, and proliferation [4,5]. Overexpression of membrane EGFR (mEGFR) has been found to correlate with poor prognosis in several cancers, including colorectal [6,7]. However, there are results that indicate that there is an independent relationship between EGFR expression and CRC prognosis [8,9]. Several studies have shown that EGFR levels are a poor predictor of response to anti-EGFR therapies. In clinical trials evaluating the efficacy of cetuximab, treatment response was not related to the levels of mEGFR expression [10,11]. On the other hand, current data suggest that nuclear EGFR (nEGFR) expression contributes to acquired resistance to cetuximab [12]. It has been shown that mEGFR can be transported from the plasma membrane to the nucleus. Thus, nEGFR has two functions, first, as transcription factor it interacts with STAT3 and E2F1 to mediate transcription of cyclin-D1, iNOS, B-Myb and Aurora Kinase A, and second in direct phosphorylation of the proliferating cell nuclear antigen [13-15]. Activated cyclin-D1 controls cell cycle, particularly in the transition from G1 to S-phase. Despite a well-established role of cyclin-D1 in cell cycle progression, previous data on cyclin-D1 and clinical outcome in colon cancer have been conflicting [16,17]. According to some studies, cyclin-D1 expression has been associated with poor and according to others with good prognosis, while most results revealed no independent prognostic value of cyclin-D1 [18,19].

At the same time, membrane and nuclear EGFR expression in CRC, according to our knowledge, has not been published so far, and the present study was conducted with the aim to explore the correlation between mEGFR, nEGFR and cyclin-D1 expression on tumor cells. We hypothesized that such investigation could have predictive value by identifying biologically and probably clinically different subsets of CRC that may have diverse response to anti-EGFR therapies. Furthermore, the methods of tissue processing and EGFR scoring systems were not homogeneous among studies, so that reproducibility of data remains a major issue for clinical application of the test. Therefore, our second aim was to observe how the scoring system for EGFR expression correlates with gene EGFR expression.

## Materials and methods

### Patients and tumor specimens

This retrospective study was performed on 135 archival formalin fixed and paraffin embedded CRC tissues from Department of Pathology, School of Medicine, University of Rijeka, Rijeka, Croatia, collected consecutively from 2007 to 2009. The investigation was approved by the Ethics Committee of the institutional review board of the Rijeka University Hospital Center.

From the representative whole tissue samples of CRC stained with hematoxylin-eosin (HE), the selected paraffin blocks were immunohistochemically analyzed for mEGFR. The representative areas with positive mEGFR immunostaining were carefully selected and marked on corresponding paraffin blocks for tissue microarray (TMA) construction. Two tissue cores, each 2 mm in diameter, were placed in a recipient paraffin block using MTA Booster OI manual tissue arrayer (Alphelys, Plaisir, France). The final TMA blocks contained 270 cores with tissue specimens. Normal liver tissue was used for slide orientation. Cores were spaced at intervals of 0.5 mm in the x- and y-axes. One section from each TMA block was stained with HE for morphological assessment. Serial sections were cut from TMA blocks for immunohistochemical staining. Five-μm thick sections were placed on adhesive glass slides (Capillary Gap Microscope Slides, 75 μm, Code S2024, DakoCytomation, Glostrup, Denmark), left to dry overnight at 37°C and stored in the dark at + 4°C.

Histological grade and stage of disease were classified according to WHO and TNM classification of Tumors of the Colon and Rectum [20].

### Immunohistochemistry and evaluation

Immunohistochemistry for mEGFR was performed on tissue section using EGFR mouse monoclonal antibody (IgG_1 _clone 2 - 18C9; pharm Dx™ ready to use kit Dako Cytomation, Glostrup, Denmark) that recognizes membrane EGFR in paraffin-embedded tissue (antibody is raised against the NH2 terminus). According to the manufacturer's instructions, for EGFR staining we employed antigen retrieval using proteinase K. For cyclin-D1 we used clone SP4 (Dako Cytomation, Glostrup, Denmark), and for nEGFR mouse monoclonal antibody IgG1 clone EGFR.25, 1:400 (Leica Biosystems, Newcastle Ltd., UK) (antibody is raised against the COOH terminus and recognizes both membrane and nuclear EGFR). Standard immunohistochemistry procedure was performed on a Dako Autostainer Plus (DakoCytomation, Colorado Inc, Fort Collins, CO, USA) according to the manufacturer's instructions, using appropriate DakoREAL solutions (Dako, Glostrup, Denmark). For negative control, an irrelevant murine monoclonal IgG antibody was used (DakoCytomation). As positive control for cyclin-D1 we used cervix (glandular cervical mucosa of endocervix) and for nEGFR liver tissue with positive nuclear staining of hepatocytes.

Scoring system was used for all three immunohistochemical markers. With this method, the intensity of immunohistochemical reaction and the proportion of positive tumor cells were recorded. Membrane EGFR was assessed according to the intensity of immunoreactivity and percentage of positive tumor cells, as follows: 0, no immunoreactivity; 1, weak and incomplete staining; 2, moderate complete and basolateral membrane immunoreactivity; and 3, strong complete and basolateral membrane immunoreactivity; the percentage was ranked as 0, negative or no immunoreactivity; 1, 1%-25%; 2, 26%-50%; and 3, 51%-100% of neoplastic cells positive. The intensity of nEGFR and cyclin-D1 expression was evaluated using the following scoring approach: 0, no immunoreactivity; 1, weak brown staining nuclei of neoplastic cells; and 2, strong brown staining nuclei of neoplastic cells; and percentage was graded as 0, negative or no immunoreactivity; 1, 1%-25%; 2, 26%-50%; and 3, 51%-100% of neoplastic cells positive.

Total score was determined by adding together the scores of staining intensity and percentage of tumor cells showing particular membrane and nuclear staining pattern (mEGFR, nEGFR, cyclin-D1), as follows: 0, negative immunoreactivity; 1-2, weak immunoreactivity; 3-4, moderate immunoreactivity; and 5-6, strong immunoreactivity.

The evaluation of immunostaining was performed by two pathologists (A.D. and N.J.). Accuracy of the immunostaining analysis was calculated based on the interobserver agreement of 100%. For statistical analysis, the mean value of immunohistochemical staining of two TMA was used.

### Fluorescent in situ hybridization

The EGFR gene status evaluation was performed by fluorescent *in situ *hybridization (FISH) on 3-4 μm thick tissue sections. Tissue sections were treated using Paraffin Pretreatment reagent kit (Vysis, Downers Grove, USA) according to the manufacturer's instructions. Dualcolor FISH assay was performed using LSI EGFR SpectrumOrange/CEP7 SpectrumGreen probe (Abbott, Vysis, Downers Grove, IL, USA), which hybridizes to the EGFR gene (orange signal) and to the centromeric region of chromosome 7 (green signal). Briefly, TMA sections were deparaffinized in xylene substitute, rinsed in 100% ethanol and air dried. Subsequently, the slides were incubated in 0.2 N HCl for 20 minutes, rinsed in 2 × SSC, pH 7.0 and immersed for 30 minutes in 1 M NaSCN solution prewarmed at 80°C. After proteinase digestion, slide denaturation (95°C for 5 minutes) and hybridization (37°C overnight) were carried out in HYBrite™(Vysis, IL, USA). On the next day, the slides were washed, counterstained with DAPI (Vysis, Downers Grove, IL, USA) and examined under fluorescent microscope (Olympus BX50, Tokyo, Japan).

The EGFR gene assessment of *in situ *hybridization was carried out by counting gene specific signals and corresponding control signals in 20 nuclei, at two areas of colorectal carcinoma. Overlapping or damaged nuclei were disregarded. A cell with a normal number of copies of the EGFR gene or chromosome 7 status was characterized by 2 orange and 2 green signals *per *nucleus. A tumor was characterized as polysomic if there were more than two CEP7 signals *per *cell in more than 40% of tumor cells or the ratio was 2.5-2.93, and amplification of EGFR was defined as the presence of the oncogene/centromere ratio ≥ 2.94 [21].

### Statistical analysis

The data collected were statistically evaluated using the data analysis software system STATISTICA, version 8.0 StatSoft, Inc. The categorical and counting variables were presented by frequencies and percentages. The possible association of variables was analyzed using regression analysis, where correlation of variables was presented by Spearman rank correlation coefficient. The level of statistical significance was set at 0.05 in all analyses.

## Results

### Expression of EGFR and cyclin-D1 in comparison with clinicopathologic features in colorectal carcinoma

**Figure 1:**
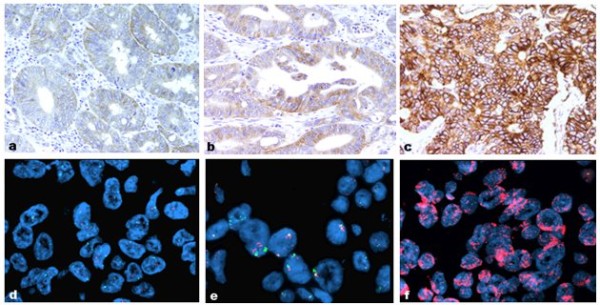
**EGFR protein and gene status in colorectal carcinomas**. Immunohistochemical expression of EGFR protein designated as discontinuous, basolateral or continuous membrane staining of different intensity: **(a) **week, **(b) **moderate and **(c) **strong immunostainining. Chromosome 7 copy number was analyzed in tumor cells using fluorescence in situ hybridization (FISH) with an α-satellite DNA probe for chromosome 7 (centromere 7, red signal; EGFR gene, green signal); **(d) **tumor nuclei without abnormality; **(e) **tumor cells showed polysomy with a greater number of red and green signals than in normal cells; and **(f) **tumor cells with amplification (a-c, magnification ×200, d-f magnification ×100).

Among 135 CRCs analyzed for EGFR, we detected strong membrane staining in 22 (16.3%) and strong nuclear staining in 77 (57%) cases. Sixty-four (47.4%) cases had moderate membrane and 49 (36.3%) moderate nuclear staining, while 47 (34.8%) had low or undetectable mEGFR and 9 (6.7%) low or undetectable nEGFR staining. In total, 135 tumor samples were examined and classified into three cyclin-D1 expression groups. Eight (5.9%) tumors were classified as negative or weakly positive, 51 (36.3%) as moderate, and 76 (57%) as strong expression samples. Finally, out of 135 tumor samples, 8 (5.9%) showed amplification of EGFR gene, 10 (7.4%) demonstrated polysomy, and 117 (86.7%) had no changes of the EGFR gene. The staining pattern characteristics of tumors with mEGFR scored weak, moderate and strong, and FISH EGFR findings are shown in Figure 1.

**Figure 2:**
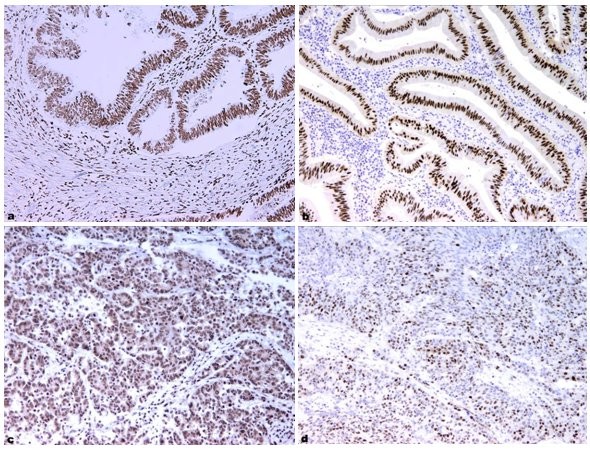
**Immunohistochemical expression of nucelar EGFR (nEGFR) and cyclin-D1 in colorectal carcinoma**. Low (a, b) and high (c, d) grade adenocarcinoma with nEGFR (a, c) and cyclin-D1 (b, d) expression.

The results of correlation between clinicopathologic features, which include histologic grade of adenocarcinoma, tumor size and stage, and immunohistochemical markers as well as FISH outcome are summarized in Table 1. Statistically significant difference was only found between tumor grade and expression of mEGFR (*p *= 0.042). In particular, high grade tumors were mostly characterized with moderate mEGFR expression, and low grade CRC with weak mEGFR expression. On the contrary, nEGFR and cyclin-D1 expression was not associated with the clinicopathologic characteristics analyzed. Two representative cases of low and high grade CRC, both having strong nEGFR expression, are shown in Figure 2.

**Table 1:** Tumor grade, size and stage in compare to immunohistochemicals markers (mEGFR, nEGFR, Cyclin D1) and EGFR FISH in CRC

		Grade			pT					Tumor stage				
Parameters	Total	low	high	p-value	1	2	3	4	p-value	1	2	3	4	p-value
mEGFR				0.042					0.506					0.769
neg./week	49	47	2		2	35	12	0		22	4	23	0	
moderate	64	54	10		0	39	23	2		22	9	33	0	
strong	22	20	2		0	12	9	1		7	4	10	1	
nEGFR				0.427					0.452					0.943
neg./week	9	9	0		0	6	3	0		4	2	3	0	
moderate	49	44	5		2	25	20	2		15	11	22	1	
strong	77	68	9		0	55	21	1		32	4	41	0	
Cyclin D1				0.363					0.335					0.588
neg./week	8	8	0		0	7	1	0		4	1	3	0	
moderate	51	46	5		2	24	22	3		14	11	25	1	
strong	76	67	9		0	55	21	0		33	5	38	0	
EGFR FISH				0.486					0.713					0.178
≤ 2.0 - 2.49	117	106	12		2	78	34	3		47	14	56	0	
2.5 - 2.93	10	9	0		0	5	5	0		2	2	5	1	
≥ 2.94	8	6	2		0	3	5	0		2	1	5	0	

Total	135	121	14		2	86	44	3		51	17	66	1	

CRC, colorectal carcinoma

mEGFR, membrane EGFR

nEGFR, nuclear EGFR

test - Spearman rank correlation coefficient

### Correlation of gene EGFR status with protein EGFR and cyclin-D1 expression

The correlation between FISH and immunohistochemical findings for EGFR gene status and protein EGFR and cyclin-D1 expression are summarized in Table 2. As mentioned above, no relationship was observed between FISH results and clinicopathologic features. On the other hand, when tissue sections used for FISH were compared with serial sections used for immunohistochemical staining, the regions of tumor cells displaying gene amplification and strong protein mEGFR expression overlapped (*P <*0.001). More precisely, most of CRCs with EGFR gene amplification were classified in the category with strong (5/8) and only in the minority of cases in the category with moderate (3/8) mEGFR immunoexpression. On the contrary, all CRCs (49/49) with normal EGFR gene status corresponded to CRCs with negative or low mEGFR expression, while polysomy in EGFR region was mostly (8/10) associated with moderate and in rare case with strong mEGFR expression. There was no correlation of EGFR gene status with nEGFR and cyclin-D1 (*P = *0.818 and *P *= 0876, respectively).

**Table 2:** Correlation between FISH EGFR and immunohistochemicals markers (mEGFR, nEGFR and Cyclin D1)

		EGFR FISH			
**Parameters**	**Total**	**≤ 2,0 - 2,49**	**2,5 - 2,93**	**≥ 2,94**	**p - value**

mEGFR					0.001
neg./week	49	49	0	0	
moderate	64	53	8	3	
strong	22	15	2	5	
nEGFR					0.818
neg./week	9	9	0	0	
moderate	49	42	2	5	
strong	77	66	8	3	
Cyclin D1					0.876
neg./week	8	7	1	0	
moderate	51	45	1	5	
strong	76	65	8	3	

Total	135	117			

CRC, colorectal carcinoma

mEGFR, membrane EGFR

nEGFR, nuclear EGFR

test - Spearman rank correlation coefficient

### Cyclin-D1 expression in relation to immunoexpression of EGFR in CRC

**Figure 3:**
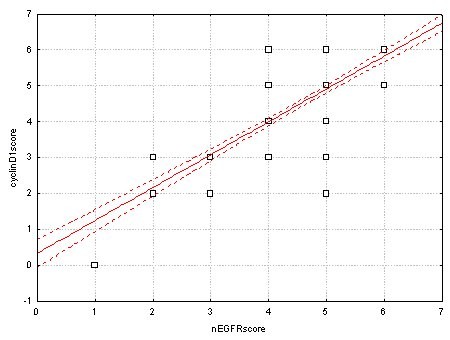
**Correlation between nuclear EGFR (nEGFR) and cyclin-D1 expression in colorectal carcinomas (r = 0.88, p < 0.001)**.

Table 3 presents the results of statistical analysis between immunoexpression of membrane and nuclear EGFR and cyclin-D1 expression. There was no association between mEGFR and cyclin-D1 (*P = *0.672), while nEGFR expression was strongly related to cyclin-D1 expression. More precisely, strong cyclin-D1 was mostly seen in CRC with strong nEGFR (*P <*0.001) (Figure 3).

**Table 3:** Immunoexpression of Cyclin D1 in relation to EGFR in CRC

		Cyclin D1			
**Parameters**	**Total**	**neg./week**	**moderate**	**strong**	**p - value**

mEGFR					0.672
neg./week	49	5	19	25	
moderate	64	3	21	40	
strong	22	0	11	11	
nEGFR					< 0.001
neg./week	9	6	3	0	
moderate	49	1	42	6	
strong	77	1	6	70	

Total	135	8	51	76	

CRC, colorectal carcinoma

mEGFR, membrane EGFR

nEGFR, nuclear EGFR

test - Spearman rank correlation coefficient

## Discussion

In previous immunohistochemical studies, there was considerable discrepancy in the frequency and distribution of EGFR overexpression in CRC. Many of these studies gave inconclusive information on the association of the protein expression and clinicopathologic features [8-11]. Furthermore, in clinical trials evaluating the efficacy of cetuximab, treatment response was not related to the levels of EGFR expression, since many patients with EGFR expressing CRC failed to respond [10,11], or those with EGFR negative tumor responded to therapy [12]. Several technical reasons have supported the lack of this association, such as prolonged storage, tissue-fixation methods, the antibodies used, the detection techniques and criteria used on result evaluation. In addition, according to some authors, the possible reasons for EGFR levels being a poor predictor of response to anti-EGFR therapies include disparity between the form or epitope of EGFR detected by immunohistochemistry and the one targeted by anti-EGFR monoclonal antibodies [22]. Recent emerging data suggest the existence of a new mode of EGFR signaling pathway in which activated EGFR undergoes nuclear translocation, and based on *in vitro *study nEGFR may play a functional role in the response to molecular therapeutic agents [12]. These intriguing findings emphasize the relevance of evaluating both the membrane and nuclear EGFR expression in CRC in order to provide more independent information on the protein association with clinicopathologic features. The present study, to our knowledge, is the first with concurrently examined mEGFR and nEGFR expression in the same cohort of CRC patients. The results confirmed the heterogeneity in mEGFR and nEGFR expression without any correlation between these proteins. Clinical significance of the present findings needs further investigation.

In our study, strong mEGFR was demonstrated in less than 20% of CRC cases, while other studies report on EGFR overexpression in up to 82% of cases [23]. Along with different methodologies used, other studies reporting a higher percentage of mEGFR expression also used less strict criteria in defining membrane overexpression. In the present analysis, only tumors with moderate to strong complete membrane staining in more than 50% of tumor cells were interpreted as strong expression, which is probably the major cause of such a low prevalence of mEGFR overexpression. Strong mEGFR expression found in our study was associated with gene amplification, found in approximately 6% of CRC samples, while moderate mEGFR was observed in those tumors that showed chromosome 7 polysomy. The result obtained is in agreement with studies where EGFR amplification has been reported to correlate with expression, although the authors stress that amplification does not reliably predict EGFR overexpression, or that overexpression of EGFR caused by amplification comprised only a small portion of the cells in these tumors [22]. The relatively low prevalence of strong mEGFR expression (overexpression) associated with the prevalence of EGFR gene amplification in CRC samples supports the evaluation of immunohistochemical staining used in our study as probably being more objective. However, in our cohort of CRCs, the strong nEGFR expression did not correlate either with strong mEGFR or with EGFR gene amplification, suggesting that gene amplification is probably not a significant event that would lead to higher nuclear translocation of the EGFR membrane receptor, as found in breast cancer model [24].

In comparison to clinicopathologic characteristics, the only significant association was found between mEGFR expression and histologic grade of CRC, or more precisely, low grade carcinomas were more characterized with negative or weak mEGFR expression. This observation was also demonstrated in other studies, where an association with more advanced stage, lymphovascular invasion, and poor prognosis was also found [6,7].

Strong nEGFR expression was confirmed in more than 50% of cases and strong cyclin-D1 expression in CRC samples was obtained in nearly the same percentage. There are no studies on the clinical significance of nEGFR expression in CRC, except for those referring to the breast, oropharyngeal squamous and ovarian cancer [14,24-26], with the expression associated with worse prognosis [12,24,25], increased proliferation and cyclin-D1 expression [25]. In the present study, nEGFR was associated with cyclin-D1. The clinical significance of these findings should be further investigated. Yet, it is important to discuss the finding reported by Li *et al. *[12] that HER family ligands are up-regulated in the cells with acquired resistance to cetuximab. The authors conclude that nEGFR may prove a viable molecular target, and according to our findings we presume that these CRC could be immunohistochemically recognized.

In our study, strong cyclin-D1 expression was observed in 57% of CRC cases. Those CRC with strong cyclin-D1 expression showed positive correlation with nEGFR, as also found in breast cancer [25]. According to previous studies, increased cyclin-D1 expression occurs in one-third of colonic tumors as an early event during the multistage process of colon carcinogenesis [16]. Although some studies demonstrated cyclin-D1 as an independent indicator of poor prognosis [17], a large cohort study suggests that cyclin-D1 expression is independently associated with good prognosis in colon cancers It is very common to assume that oncogene activation (or tumor suppressor inactivation) is associated with aggressive tumor behavior. However, as commented by the authors, this hypothesis does not always hold true, since it is well recognized that MSI, which is known to cause inactivation of a number of tumor suppressors (including TGFBR2, BAX, and many others), is associated with better patient outcome [27].

## Conclusions

Standardized chemotherapy regimens result not only in improved efficacy but also in an increased toxicity and treatment costs, therefore requiring identification of decision-making tools to select patients who are likely to benefit from them. Both KRAS mutation and EGFR disomy represent two negative-predictive factors able to impair clinical responsiveness to anti-EGFR therapy with monoclonal antibodies [28-30]; however, our findings indicate the importance of further investigation of standardized protocols for immunohistochemical analysis of both mEGFR and nEGFR, which will be applicable in clinical practice.

## Competing interests

The authors declare that they have no competing interests.

## Authors' contributions

AD participated in the design of the study and drafted the article. AD and SP participated in collection of references and evaluation of the data. RDD and IB participated in collection of the data. MŽB performed the statistical analysis. NJ participated in the design, coordination and reviewing the manuscript. All authors read and approved the final manuscript.
